# Pressure-Induced Modulation of Tin Selenide Properties: A Review

**DOI:** 10.3390/molecules28247971

**Published:** 2023-12-06

**Authors:** Ziwei Cheng, Jian Zhang, Lin Lin, Zhiwen Zhan, Yibo Ma, Jia Li, Shenglong Yu, Hang Cui

**Affiliations:** 1 College of Sciences, Beihua University, Jilin 132013, China; chengziwei1102@126.com (Z.C.); zhanzhiwen2021@126.com (Z.Z.); mayibo_beihua@126.com (Y.M.); lj06407@163.com (J.L.); yushenglong88@163.com (S.Y.); 2 School of Agricultural Engineering and Food Science, Shandong University of Technology, Zibo 255000, China; linlin_beihua@126.com; 3 Key Laboratory of Wooden Materials Science and Engineering of Jilin Province, Beihua University, Jilin 132013, China; 4 State Key Laboratory of Superhard Materials, College of Physics, Jilin University, Changchun 130012, China; cuihang@jlu.edu.cn

**Keywords:** SnSe, crystal structure, X-ray diffraction, high pressure, phase transition

## Abstract

Tin selenide (SnSe) holds great potential for abundant future applications, due to its exceptional properties and distinctive layered structure, which can be modified using a variety of techniques. One of the many tuning techniques is pressure manipulating using the diamond anvil cell (DAC), which is a very efficient in situ and reversible approach for modulating the structure and physical properties of SnSe. We briefly summarize the advantages and challenges of experimental study using DAC in this review, then introduce the recent progress and achievements of the pressure-induced structure and performance of SnSe, especially including the influence of pressure on its crystal structure and optical, electronic, and thermoelectric properties. The overall goal of the review is to better understand the mechanics underlying pressure-induced phase transitions and to offer suggestions for properly designing a structural pattern to achieve or enhanced novel properties.

## 1. Introduction

Tin selenide (SnSe) has attracted considerable attention owing to its layered structure, narrow bandgap, anisotropy, and other advantages such as chemical stability, low toxicity, and natural abundance [1,2,3,4,5,6,7,8]. The orthorhombic structure of SnSe exhibits beneficial properties for two-dimensional (2D) growth and possesses a direct gap of 1.3 eV and an indirect gap of 0.9 eV [9,10]. Moreover, SnSe also exhibits considerable potential in the fields of thermoelectricity, holographic recording, photocatalysis, memory switching, infrared electronics, and energy storage [9,10,11,12,13,14,15,16]. The interlayer degree of freedom can be manipulated to effectively control its properties and the interlayer interaction exhibits a significant correlation with external factors, including pressure, temperature, strain, and electromagnetic field. These disruptions offer a variety of methods for altering SnSe properties [17,18,19,20,21,22,23,24,25].

It is well known that the physical and chemical properties of materials are extraordinarily dependent on its crystal structure [26]. The applications of the high-temperature-phase *β*-SnSe are limited due to its phase transition temperature of up to 800 K, despite its thermoelectric performance resulting from its highly symmetric structure [27,28,29,30,31]. Pressure plays a distinctive and advantageous role due to its ability to alter the interlayer interaction, resulting in the exploration and production of novel materials and properties, while maintaining constant stoichiometry and temperature. However, the effect of pressure on the preparation and performance of SnSe is frequently underestimated or even overlooked.

This review aims to provide a comprehensive overview of the recent progress and achievements of pressure-induced structure and properties in SnSe. Section 2 provides a comprehensive explanation of high-pressure techniques, focusing on diamond anvil cells (DACs), and an exploration of diverse characterization tools that can be integrated with DACs to effectively monitor the in situ changes in the structure and properties of materials under high-pressure conditions. Section 3 analyzes the influence of pressure on the crystal structure of both bulk and nanoscaled SnSe and explains the distinctions and similarities in the phase transition behavior exhibited by these materials. Section 4 presents the effect of pressure on the performance of SnSe, including its optical, electronic, and thermoelectric properties. The overview and perspective are concluded in Section 5. This review attempts to improve understanding of the pressure-induced structural transition of SnSe and provide valuable insights for future investigations on the development of structurally planned patterns.

## 2. High-Pressure Techniques

The synthesis of materials is primarily governed by the concepts of kinetics and thermodynamics during the chemical synthesis process. Conversely, under high-pressure conditions, an intriguing opportunity arises to manipulate the phase or structure of materials. Pressure is an essential thermodynamic parameter that can be used to control the properties of materials. By decreasing interatomic distances and altering electronic orbitals and bonding structures, pressure serves as a versatile mechanism for generating unique materials that are typically impossible to generate at ambient conditions [32,33,34,35]. This arises due to the free-energy alteration within the material system, attributed to the *PV* (*P* is the pressure, *V* is the volume) term in the Gibbs free energy (*G*). Currently, high-pressure conditions or environments can be realized through several methods, including the high-pressure piston-cylinder [36,37,38,39], cubic anvil cell [40,41,42], and diamond anvil cell (DAC) [43,44,45]. Among these techniques, the DAC technology stands out as a potent and versatile tool for generating ultra-high pressure and facilitating the measurement of small samples.

### 2.1. DAC Technology

**Figure 1:**
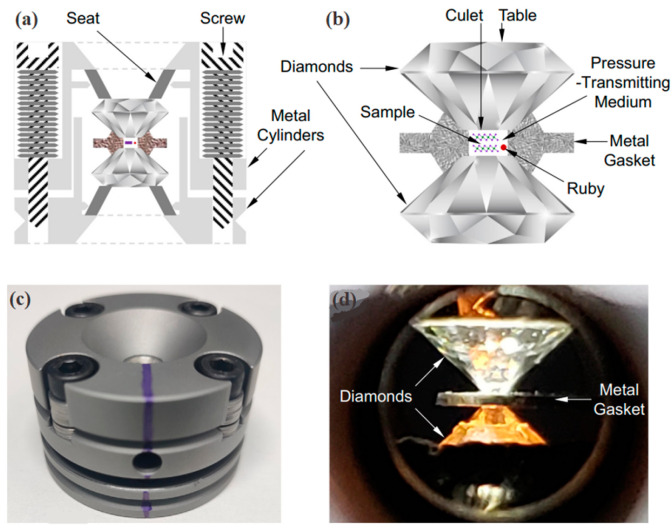
Schematic diagrams (**a**,**b**) and images (**c**,**d**) of a typical DAC. Reprinted with permission from Ref. [46]. Copyright 2022, Elsevier.

With well-configured installation, DAC demonstrates the remarkable capability to generate pressures as high as 550 GPa. It surpasses the pressure at the core of the Earth, indicating that DAC has the potential to replicate the majority of pressure-induced processes occurring on Earth. This exceptional tool has gained global popularity due to its compact size, ease of operation, and safety. As shown in Figure 1, DAC consists of a pair of diamond anvils along with external mechanical components [46]. The diamond possesses a unique combination of mechanical, thermal, electrical, optical, chemical, and other qualities, making it an outstanding choice for an anvil.

A sealing gasket is positioned between the two anvil faces. The gasket features a cylindrical hole at its center, serving as a dedicated sample chamber. The sealing gasket has two functions: it alters the stress distribution and seals the sample. Gasket materials include T301 steel, rhenium, titanium, and copper. To ensure a uniform compression of the sample within the DAC, pressure mediums are essential for transmitting pressure and maintaining a hydrostatic pressure field. Pressure transmitting mediums generally follow specific requirements, including no chemical reactivity with the sample, efficient pressure transmission, and good temperature stability. Table 1 provides a comprehensive overview of various pressure-transmitting materials, highlighting the unique characteristics related to each group. Monitoring and calibrating the applied pressure is a crucial step in high-pressure experiments. Ruby is widely regarded as the ideal choice due to it exhibiting strong fluorescence when subjected to laser excitation, and its luminescence spectrum exhibits a distinct peak that consistently varies in response to applied pressure.

### 2.2. In Situ High-Pressure Measurement

In situ high-pressure X-ray diffraction (XRD) is a valuable technique for investigating material structures under extreme pressure conditions, providing compelling evidence of phase transitions [52,53]. Compared to the XRD analysis at ambient conditions, in situ XRD presents many challenges, including the limited size of the sample chamber, the absorption of X-rays by the diamond anvils, and the constrained numerical aperture of the DAC. These problems have been effectively improved with the development of synchrotron radiation technology. Meanwhile, researchers have developed devices with specific geometries to overcome the numerical aperture limitations, although these mechanical changes reduce the maximum pressure which can be achieved.

Raman spectroscopy is particularly sensitive to various factors, including the polarization and orientation of the incident light, the crystal symmetry and orientation of the solid sample, and the direction and polarization of the scattered light. Under high-pressure conditions, the in situ Raman spectroscopy technique can provide valuable insights into the strength of chemical bonds, the coefficient of thermal expansion and compression, and the chemical processes or phase transitions. In particular, this method excels in monitoring the interlayer sliding phenomena due to its ability to effectively measure the impact of pressure on the covalent bonds within the material (high-frequency Raman vibrations) and the spacing between layers (low-frequency Raman vibrations). However, it is worth noting that Raman scattering often exhibits relatively low intensity. To enhance the signal-to-noise ratio, various strategies can be used, including extending the integration time, conducting multiple scans, refining the oscillator, and implementing additional signal filtering.

## 3. Pressure-Induced SnSe Structural Transitions

**Figure 2:**
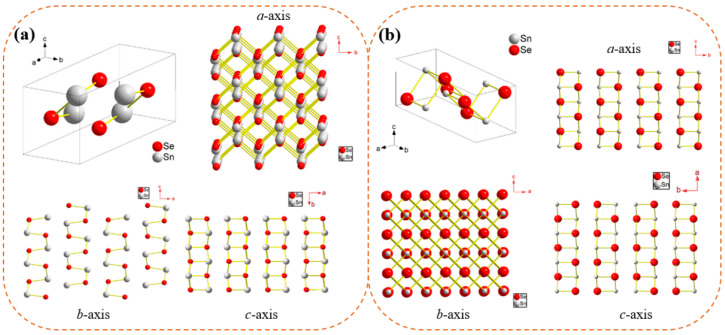
Crystal structure of (**a**) *α*-SnSe and (**b**) *β*-SnSe. Reprinted with permission from Ref. [58]. Copyright 2018, Elsevier.

During the application of pressure to substances with constant composition and temperature, a variety of materials exhibit structural transition. Structural transitions, associated with phase transitions, cell volumes, lattice parameters, and orientations, can be induced by fundamental thermodynamic variables [54,55]. Two stable structures of SnSe have been reported: *α*-SnSe (*T* < 800 K) and *β*-SnSe (*T* > 800 K). The *α*-SnSe belongs to the orthorhombic structure with *Pnma* space group and the *β*-SnSe belongs to the orthorhombic structure with the *Cmcm* space group [56]. As shown in Figure 2, it is evident that *α*-SnSe possesses a double-layered structure, which can be regarded as a three-dimensional (3D) distortion of the NaCl structure [57]. *β-*SnSe is similar to *α*-SnSe but with a higher symmetry. Meanwhile, in light of prior researches, the high-pressure behavior of SnSe bulk materials is very different from that of nanomaterials.

### 3.1. Phase Transition of Bulk SnSe

**Figure 3:**
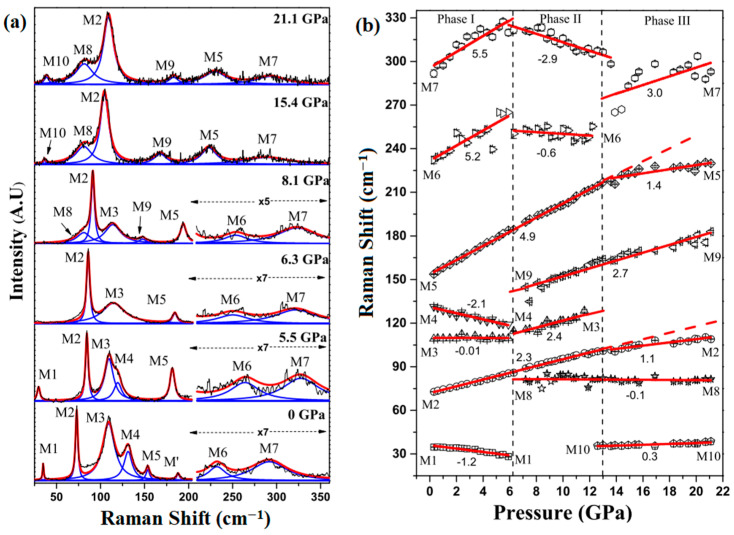
(**a**) Raman spectra of SnSe under high pressure; (**b**) the Raman shift of SnSe under high pressure. Reprinted with permission from Ref. [62]. Copyright 2020, American Physical Society.

Chattopadhyay et al. conducted a preliminary investigation via energy dispersive X-ray diffraction techniques to examine the behavior of SnSe, which revealed that the layered crystal structure exhibited stability up to a pressure of 34 GPa [59]. Peters and McNeil et al. observed structural modifications in SnSe under pressures ranging from 1.4 to 3 GPa using Mossbauer measurements [57]. These changes were attributed to the interlayer interactions, prompting the transition from a pseudo 2D structure to a 3D structure. Importantly, the structural characteristics of the *Pnma* space group were maintained during this transformation. In contrast, Alptekin et al. theoretically determined that a transition from the orthorhombic *Pnma* phase to the *Cmcm* phase occurs at 7 GPa [60]. Loa et al. utilized angle-dispersive synchrotron X-ray powder diffraction (ADXRD) to investigate the behavior of SnSe under pressure up to 10.5 GPa. They observed SnSe undergo a second-order phase transition from the orthorhombic *Pnma* structure to *Bbmm*, which is similar to *Cmcm*. This study represents the first instance of experimental evidence that supports the presence of a structural phase transition in bulk SnSe [61]. Pal et al. also observed the structural phase transition in bulk SnSe through Raman scattering. As shown in Figure 3, two new modes (M8, M9) at 81.2 and 144.6 cm^−1^ emerged in the Raman spectra at 6.2 GPa and persisted up to 21 GPa, which is associated with the phase transition [62].

**Figure 4:**
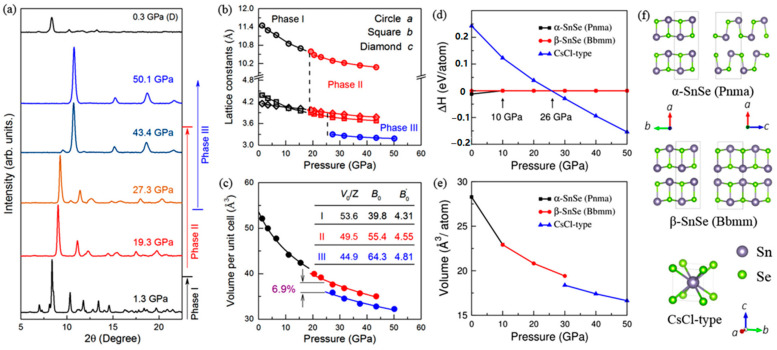
(**a**) High-pressure ADXRD of SnSe; (**b**) lattice parameters as a function of pressure; (**c**) cell volume as a function of pressure; (**d**) calculated enthalpy relative to that of the *β*-SnSe; (**e**) unit cell volume vs. pressure; and (**f**) crystal structures of *α*-SnSe, *β*-SnSe and *CsCl*-type SnSe. Reprinted with permission from Ref. [63]. Copyright 2017, American Physical Society.

In a larger pressure range (around 27 GPa), Chen and Lu et al. observed a new phase transition from the orthorhombic structure to the cubic structure in bulk SnSe [63]. Figure 4 provides evidence that the structural transitions occurring at low pressure and high pressure are classified as second-order and first-order, respectively. The second-order phase transition is caused by the displacive movement of Sn/Se atoms in the [010] direction, and the first-order phase transition was initiated by the bonding of Sn to Se from nearby bilayers. As shown in Figure 4d,e, theoretical calculations are in good agreement with experimental results. Subsequent findings by other researchers have further confirmed the existence of SnSe cubic structure at higher pressure [64,65,66].

### 3.2. Phase Transition of SnSe Nanomaterials

Nanomaterials possess distinct characteristics that largely depend on their crystal phase, surface area, morphology and architecture [30,67,68,69]. In contrast to bulk materials, nanomaterials exhibit size- and shape-dependent high-pressure phase transition behavior, including anomalous pressure responses and novel physical-chemical properties.

**Figure 5:**
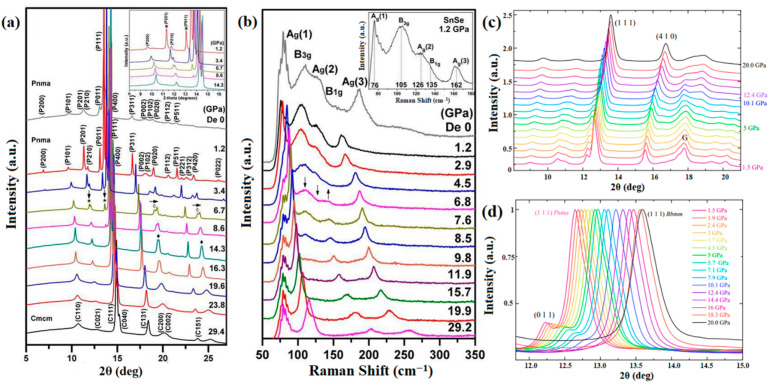
(**a**) ADXRD patterns of SnSe nanosheets; (**b**) selected Raman spectra of SnSe nanosheets under high pressure. Reprinted with permission from Ref. [70]. Copyright 2015, The Royal Society of Chemistry. (**c**) ADXRD patterns of SnS_0.5_Se_0.5_; (**d**) (111) peak shift in the function of the pressure. Reprinted with permission from Ref. [73]. Copyright 2019, Elsevier.

Zhang et al. synthesized SnSe single-crystalline nanosheets with an average thickness of 25 nm and a lateral dimension of 500 nm using the plasma assisted direct current arc discharge method [70]. As shown in Figure 5a,b, the second-order isostructural continuous phase transition from *Pnma* to *Cmcm* was observed at 6.8 GPa, which is much lower than the transition pressure of bulk SnSe [61]. The decreased transition pressure can be attributed to the volumetric expansion, with the softening of the Poisson ratio and shear modulus. This abnormal compressibility arises from the unique intrinsic geometry in the nanosheets. Meanwhile, all diffraction peaks and vibrational modes returned to the original structure when the pressure was released to the ambient pressure, indicating the pressure-induced structural transformation is reversible. Studies have found pressure-induced polymorphism in nanostructured SnSe produced by mechanical ball-milling [71,72]. Marques et al. performed mechanical ball-milling on nanostructured SnS_1-*x*_Se*_x_* (*x* = 0.5), providing further insights into the role of disorder in the pressure-induced transformation of the *Pnma* structure [73]. A nanostructured SnSe_0.5_S_0.5_ was obtained through mechanical milling of high-purity Sn (99.995%), Se (99.999%) and S (99.999%) powders. The powders were sealed together with several steel balls into a cylindrical steel vial under argon atmosphere. The ball-to-powder ratio was 5:1; a SPEX type mill was used to perform mechanical alloying at room temperature. As shown in Figure 5c,d, SnS_1-*x*_Se*_x_* (*x* = 0.5) exhibits consistent enhancement in symmetry with the *Bbmm* structure. The observed dynamics of the transition suggest an isotropic change in the lattice parameters when the critical pressure falls within the range of 10 to 14 GPa. Their theory suggests that this observed variation may be attributed to the degree of stacking defects, as layered structures prove to be highly sensitive to shear deformations. 

In order to analyze the transformation process that applies to the high-pressure behavior of SnSe, the changes in cell volume and bulk modulus are listed in Table 2. It is worth noting that SnSe nanomaterials display a lower transition pressure compared to their bulk counterparts [74,75,76,77]. The reduction in transition pressure can be attributed to the volumetric expansion with the softening of the Poisson ratio and shear modulus. Meanwhile, the bulk modulus of SnSe nanomaterials under ambient pressure coincides with that of bulk SnSe. However, the bulk modulus of SnSe nanomaterials under high pressure is much higher than that of bulk SnSe. The enhanced bulk modulus can be attributed to the higher surface energy due to the pressure-induced morphology change [78,79,80].

## 4. Properties of SnSe under High Pressure

Pressure, along with temperature, is considered one of the fundamental thermodynamic variables. It serves as a powerful tool for manipulating the structures of material systems, combining the in situ techniques, and making it possible to establish the correlations between structure and properties [81,82]. Pressure is a clean tool for structure tuning compared with other methods. For instance, the distance between particles can be adjusted by high pressure [83,84,85]. In this section, the pressure-dependent optical, electronic, and thermoelectric properties of SnSe are reviewed.

### 4.1. Optical Properties of SnSe under High Pressure

The investigation of optical characteristics is crucial to understand the internal composition of materials and assessing their potential for practical application as optoelectronic devices. It is crucial for investigating the connections between structural transition and material properties, enhancing material properties, and potentially facilitating applications under ambient pressure conditions. The study on the optical constants and properties under pressure serves as a framework for investigating the optoelectronic characteristics of SnSe.

#### 4.1.1. Optical Constants

**Figure 6:**
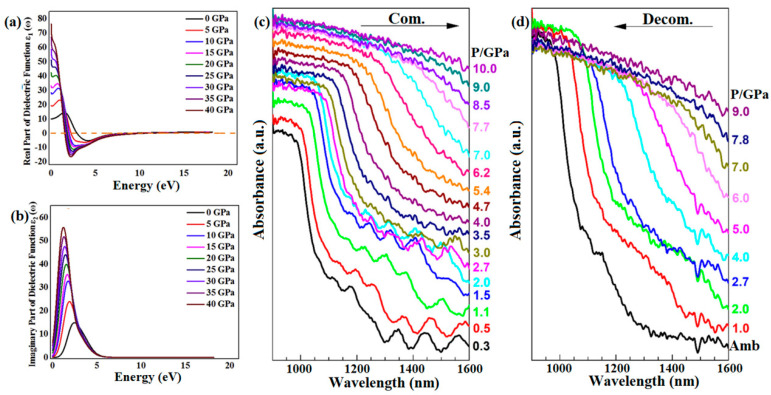
(**a**,**b**) Frequency-dependent dielectric function of π-SnSe at 0–40 GPa. Reprinted with permission from Ref. [89]. Copyright 2017 Elsevier. (**c**,**d**) NIR absorption spectra of SnSe during compression and decompression at room temperature. Reprinted with permission from Ref. [66]. Copyright 2023, AIP Publishing.

Cubic phases of SnSe (π-SnSe) were first discovered in 2015–2016 [86,87,88]. Rehman et al. performed a detailed analysis of the structural and optical properties of cubic phase π-SnSe under pressure [89]. As shown in Figure 6a,b, the dielectric constant exhibits an increasing trend with the pressure at the range of 0–40 GPa. It is clear from Table 3 that optical absorption, the dielectric constant, optical conductivity and static refractive indices follow an increasing trend as the external pressure increases from 0 to 40 GPa, and combined with the tendency of reflection and loss function as pressure increases, it can be concluded that external pressure might be the key reason to enhance the near/mid infrared light activity. Xie et al. reported that the increased pressure leads to a shift of the absorbance edge towards lower photon energy, which suggests a reduction in bandgap width [90]. Luo et al. further investigated the optical properties of SnSe under high pressure up to 10 GPa [66]. As shown in Figure 6c,d, the absorption edge shifts towards longer wavelengths as pressure increases and exhibits blue shift with decompression, indicating the pressure-induced modulation of the optical bandgap is reversible. Moreover, Biesner et al. discussed the optical spectra of doped SnSe under pressure [91]. Both lightly and heavily doped SnSe exhibit a narrowing in bandgap and an increase in low-frequency optical conductivity. It is interesting that the bandgap is not fully closed, even under the highest pressure. It is suggested that the phase transition is primarily attributed to the band shift across the Fermi level, rather than the collapse of the optical gap.

#### 4.1.2. In-Plane Anisotropy

Anisotropy refers to the phenomenon in which a substance exhibits directional dependence, resulting in variations in its chemical and physical properties along different axes. Understanding material anisotropy is valuable for optimizing device performance. The utilization of anisotropic features is important in the advancement of innovative technologies, including multi-channel sensors, anisotropic logic manipulation devices, and polarimetric photodetectors. The optical, vibrational, thermal, and electronic properties of layered SnSe are characterized by significant in-plane anisotropy along the orthorhombic directions, resulting from variations in bond lengths and angles [92,93,94,95,96].

**Figure 7:**
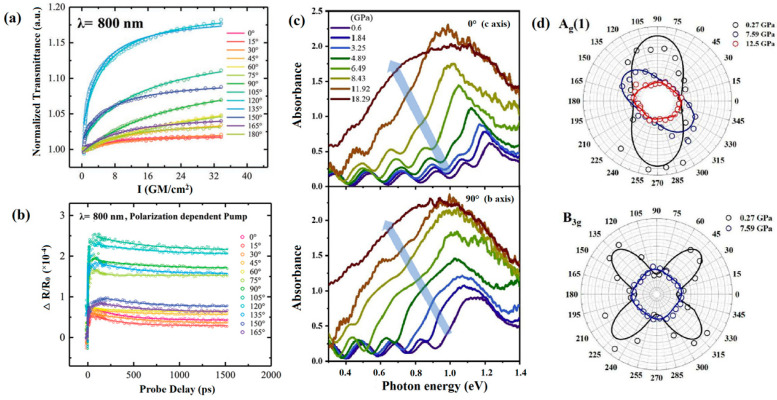
In-plane anisotropy in SnSe. Polarization-dependent (**a**) saturable absorption and (**b**) transient absorption in SnSe. Reprinted with permission from Ref. [92]. Copyright 2019, Wiley-VCH. (**c**) Pressure dependence of absorbance spectra of SnSe in directions of *b*-axis and *c*-axis; (**d**) intensity of A_g_(1) and B_3g_ modes as a function of the excitation polarization direction. Reprinted with permission from Ref. [90]. Copyright 2020, Elsevier.

Shi et al. observed a significant difference in the light absorption spectra between monolayer and few-layer SnSe, which is due to the in-plane anisotropy [93]. Zhang et al. investigated the anisotropic nonlinear optical properties of SnSe nanosheets [92]. The polarization of incident light at 800 nm had a significant impact on the linear absorption rate. As shown in Figure 7a,b, SnSe displayed anisotropy in its polarization-dependent saturable absorption and transient absorption. The analysis of transient absorption anisotropy indicates that the highest level of injected carrier density was observed when the pump polarization was aligned with the armchair direction, which can be attributed to the maximum absorption rate observed at this specific polarization. Xie et al. reported that the application of pressure leads to varying effects on optical absorption at different orientations or directions [90]. As shown in Figure 7c,d, in comparison to the *b*-axis, the absorbance peaks in the *c*-axis undergo a downward shift towards lower energy regions as pressure increases. The *c*-axis curve is similar to *b*-axis when the pressures up to 12 GPa, indicating that the anisotropy of the structure gradually decreases. In addition, the variation in the intensity of A_g_(1) and B_3g_ modes under pressure also demonstrated a similar tendency, which is clearly depicted by the rotation of the polarized plot. This change corresponded with alterations in the infrared absorption, indicating that the anisotropic optical properties of SnSe decrease with increasing pressure and disappear at 12 GPa. It also supports that SnSe undergoes a structural transition from low-symmetry *Pnma* phase to high-symmetry *Bbmm* structure at 12 GPa. The elucidation of anisotropic optical characteristics in SnSe offers valuable insights into its phase transition behavior, holding potential in the development of novel optical components, transistors, and photodetectors.

### 4.2. Electronic Properties of SnSe under High Pressure

High pressure has proven to be a powerful tool in the modulation of electronic properties, leading to alterations in the electronic structure and electrical transport characteristics of materials. The influence of pressure on the electronic properties of SnSe was discussed in terms of electronic structure and electrical transport.

#### 4.2.1. Electronic Structure

**Figure 8:**
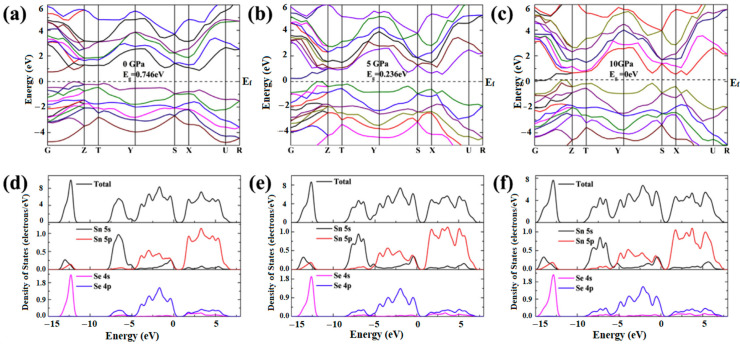
(**a**–**c**) Band structures and (**d**–**f**) DOS of SnSe under pressure. Reprinted with permission from Ref. [108]. Copyright 2016, Elsevier.

**Figure 9:**
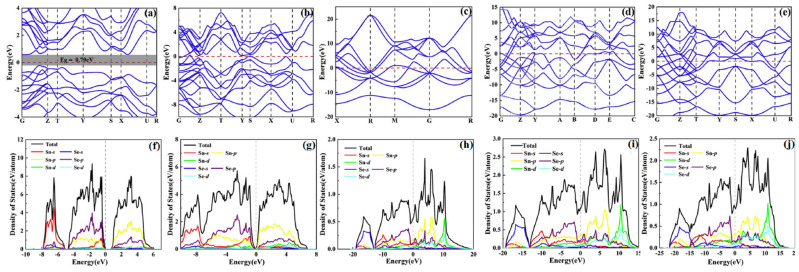
Electronic band structure of SnSe at (**a**) 0, (**b**) 22, (**c**) 78.5, (**d**) 89 and (**e**) 220 GPa, respectively; DOS of (**f**) *Pnma*, (**g**) *Cmcm*, (**h**) *Pm*$\bar{3}$*m*, (**i**) *C2/m*, and (**j**) *Cmmm* phases of SnSe. Reprinted with permission from Ref. [110]. Copyright 2021, Elsevier..

In the 1990s, optical absorption measurements revealed that the optical bandgap for *α*-SnSe was 0.923 eV [97]. As computational science rapidly advances, theoretical simulations have reported the values for the bandgap of both *α*-SnSe and *β*-SnSe: the bandgap of *β*-SnSe is only half that of *α*-SnSe [98,99,100,101,102,103,104,105,106,107]. In addition to studying the electronic structures of SnSe under ambient conditions, the electronic properties of SnSe under high pressure have been investigated. Zhang et al. used the CASTEP software package for geometric optimization and band structure calculations on SnSe within a pressure range of 0–11 GPa. Their study offers a comprehensive analysis of the pressure-induced variations in the band structure of SnSe [108]. As shown in Figure 8, the increased pressure leads the extension and movement of valence band maximum (VBM) and conduction band minimum (CBM) closer to the Fermi level, resulting the bandgap in decreasing and even in close. With the increasing pressure, the electronic states near the Fermi surface approach the Fermi level and the values of the total Density of States (DOS) gradually decreased, corresponding to the changes in the band structure features. In addition, a transition of SnSe from the semiconductor to semimetallic state occurred at 10 GPa. It can be attributed to the increased hybridization of Sn-5s, Sn-5p, and Se-4p orbitals under pressure [109]. Yang et al. investigated the influence of pressure on the electronic properties of SnSe crystals combined the CALYPSO with first-principle calculations [110,111]. Above 22 GPa, new stable structures identified as *Pm$\bar{3}$m*-, *C2/m*-, and *Cmmm*-SnSe were found. As shown in Figure 9, the energy band structures and DOS revealed that only the *Pnma*-SnSe phase was a semiconductor with a bandgap of 0.79 eV. In contrast, the other phases exhibited metallic properties.

#### 4.2.2. Electrical Transport

Electrical transport properties represent the macroscopic behaviors resulting from the intricate interactions of free electrons within materials. These interactions cause variations in the band and electronic structures, and then influence the Seebeck coefficient and electrical conductivity. Recent research has indicated that pressure presents a unique approach for optimizing the behavior of electrical transport properties in various thermoelectric materials [112,113,114].

**Figure 10:**
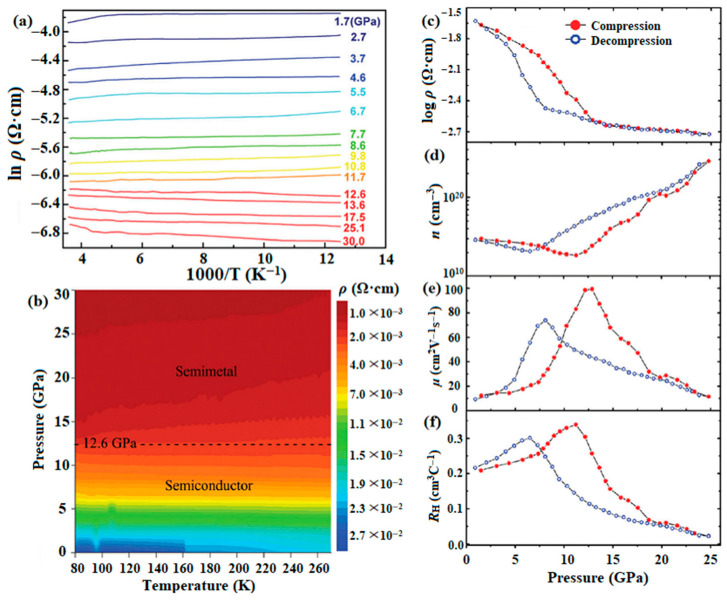
(**a**) Resistivity of SnSe at various pressures; (**b**) temperature–pressure contour plot of resistivity; (**c**) electrical resistivity, (**d**) carrier concentration, (**e**) carrier mobility, and (**f**) hall coefficient of SnSe as a function of pressure at room temperature. Reprinted with permission from Ref. [116]. Copyright 2016, The Royal Society of Chemistry.

Agarwal et al. observed an abrupt decrease in the electrical resistivity of SnSe monocrystals in the pressure range of 6 to 7 GPa [115]. As shown in Figure 10a,b, the decreased electrical resistivity is attributed to the change from a semiconductor to semimetal, which is primarily driven by a pressure-induced structural transition [116]. Yan et al. performed in situ high-pressure Hall effect measurements. As shown in Figure 10c–f, SnSe transfers into a semimetallic state at around 12 GPa. It is accompanied by the phase transition from orthorhombic to monoclinic, resulting in the alterations of carrier concentration and mobility [116].

**Figure 11:**
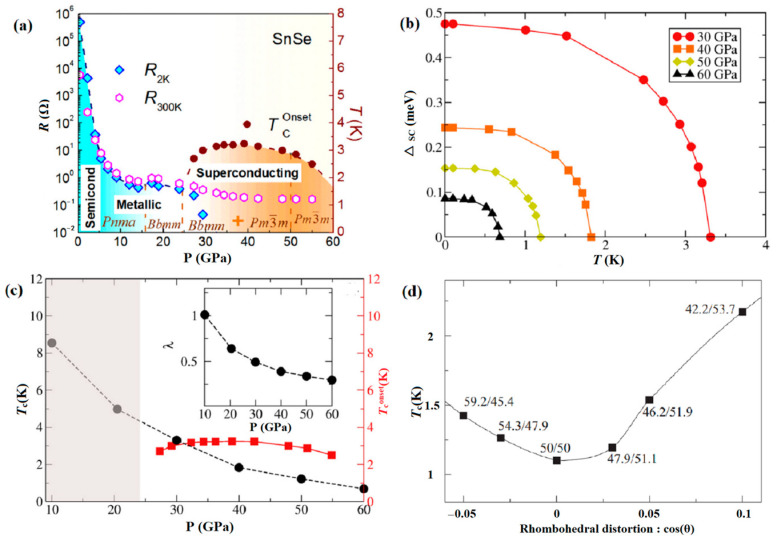
(**a**) Temperature versus pressure phase diagram. Reprinted with permission from Ref. [63]. Copyright 2017, American Physical Society. (**b**) Superconducting gap (△_SC_) as a function of the temperature at various pressures; (**c**) critical temperature of the *CsCl*-type phase as a function of the pressure; (**d**) superconducting critical temperature as a function of the angle at 50 GPa. Reprinted with permission from Ref. [117]. Copyright 2019, American Physical Society.

Chen et al. and Marini et al. subsequently confirmed the presence of a superconducting (SC) phase [63,117]. Although most reports agree that the SC phase is the *CsCl*-type structure, some inconsistency regarding the pressure range at which superconductivity emerges and the transition temperature *T*_c_(*P*). Timofeev et al. reported the SC phase of SnSe with a transition temperature *T*_c_(*P*) of 4.5 K at 58 GPa [118]. In contrast, as shown in Figure 11a, Chen observed that the SC phase initiates at 27 GPa with *T_c_* of 3 K, reaching its peak value of 3.2 K at a pressure of 39 GPa. Meanwhile, theoretical predictions revealed interesting topological features in the metallic *CsCl*-type phase. Marini et al. focused on the electronic features of the topological *CsCl*-type phase and analyzed its dynamical and SC properties. Their predictions indicated a decline in the SC transition temperature with the pressure increasing, which is in contrast with the experimental result (Figure 11b,c). It is attributed to the low value of *T*_c_(*P*) and the differences between the onset and zero-resistance [63,119]. Furthermore, additional investigations into the impact of non-hydrostatic pressure conditions on the SC critical temperature revealed that structural deformation at 50 GPa influenced *T*_c_(*P*) (Figure 11d). In addition, sulfur doping has proven effective in enhancing the SC properties of SnSe and extending the pressure range of SC over 70 GPa.

### 4.3. Thermoelectric Properties of SnSe under High Pressure

For decades, the primary strategies for enhancing the thermoelectric figure of merit (*ZT*) have been the adjustment of carrier concentration to improve the power factor and the reduction in thermal conductivity [120,121]. SnSe has demonstrated an exceptionally high *ZT* value of 2.6 ± 0.3 at 923 K [29]. However, *ZT* value remains low below 800 K, limiting practical efficiency. Significant progress has been achieved through the application of various external factors, such as pressure or stress, to enhance the *ZT* value in existing thermoelectric materials [122,123,124,125].

#### 4.3.1. Anisotropic Thermoelectric Properties

In the early days, considerable attention was drawn to the remarkable thermoelectric properties of SnSe [58,126]. Zhao et al. reported remarkably elevated *ZT* values for single-crystalline SnSe at 923 K [29]. It was as high as 2.6 ± 0.3 along the *b*-axis, 2.3 ± 0.3 along the *c*-axis, and 0.8 ± 0.2 along the *a*-axis. Carrete et al. observed the lattice thermal conductivity (κ_l_) with κ_l_^b^ > κ_l_^c^ > κ_l_^a^ at all temperatures by theoretical calculation [127]. Ibrahim et al. reported that κ_l_ presents an expressive anisotropy among the three directions (κ_l_^c^ > κ_l_^b^ > κ_l_^a^) in undoped single-crystalline SnSe [128].

**Figure 12:**
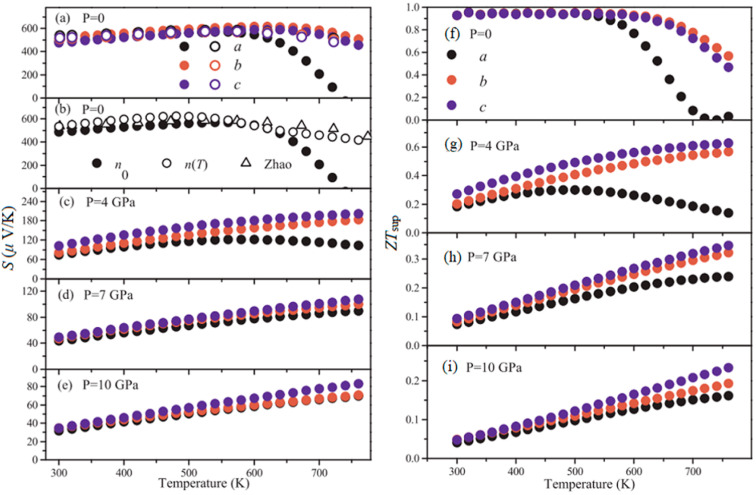
(**a**) The Seebeck coefficient at ambient pressure; (**b**) the Seebeck coefficient for the *a*-axis taking into account the dependence of the carrier concentration *n*(*T*) on temperature, for constant carrier concentration *n*_0_ (solid circle); in (**c**–**e**) the Seebeck coefficient for P = 4, 7 and 10 GPa are shown. *ZT* as a function of temperature for pressures (**f**) P = 0, (**g**) P = 4, (**h**) P = 7, and (**i**) P = 10 GPa. Reprinted with permission from Ref. [130]. Copyright 2018, Elsevier.

**Figure 13:**
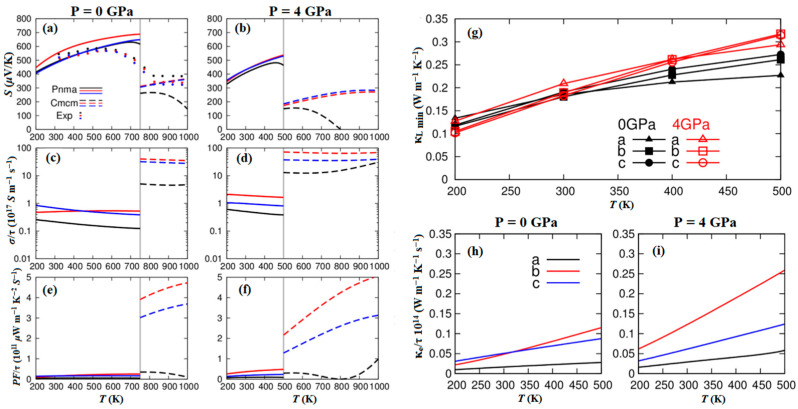
(**a**,**b**) Seebeck coefficient, (**c**,**d**) electrical conductivity and (**e**,**f**) power factor of SnSe under different pressures; (**g**) theoretical lattice thermal conductivity of *Pnma* at different pressures; electronic thermal conductivity of the *Pnma* phase as functions of temperature at (**h**) 0 GPa and (**i**) 4 GPa. Reprinted with permission from Ref. [132]. Copyright 2016, Springer Nature.

Zhang et al. predicted the *ZT* values of P-type SnSe along the *b*- and *c*-axis can reach 2.5 and 1.7 at 6 GPa and 700 K, respectively. However, the *a*-axis showed N-type SnSe with *ZT* value of 1.7 at 6 GPa and 600 K. The improvement in thermoelectric properties can be attributed to the alteration of electronic structures under pressure [129]. Gusãmo et al. investigated the impact of pressures (0–10 GPa) on the thermoelectric properties of *Pnma*-SnSe [130]. As shown in Figure 12a–e, the Seebeck coefficient exhibited a continuous decrease along the *a*-axis, which attributes this rapid decrease and signal modification to the increasing thermally excited negative electrons owing to the decrease in the energy band gap with P-type SnSe [131]. In Figure 12f–i, it is evident that the *ZT* decreases with increasing pressure, signifying that external pressure has a diminishing effect on the efficiency of the conversion of thermal energy to electrical energy. Yu et al. reported the anisotropy and thermoelectric parameters of the *Pnma* and *Cmcm* phases exhibit notable differences [132]. As shown in Figure 13, the overall effect of 4 GPa pressure on the power factor is small, but it does not significantly increase the lattice thermal conductivity, which is conducive to the enhancement of *ZT* value.

#### 4.3.2. Doping-induced Enhancement of Thermoelectric Properties

Doping is a process that involves introducing small quantities of other elements or compounds into a material, in order to enhance their properties or to induce specific electrical, thermoelectric, and optical characteristics.

**Figure 14:**
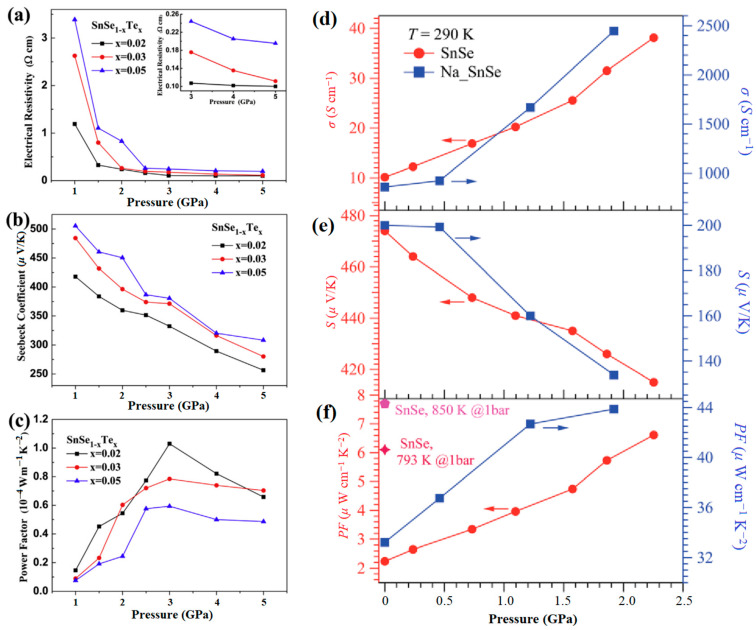
Pressure dependence of thermoelectric properties of SnSe, (**a**–**c**) electrical resistivity, Seebeck coefficient, power factor. Reprinted with permission from Ref. [108]. Copyright 2016, Elsevier. Pressure dependence of (**d**) electrical conductivity, (**e**) Seebeck coefficient, (**f**) power factor at 290 K for pristine SnSe (**left**) and Na-doped SnSe (**right**). Reprinted with permission from Ref. [137]. Copyright 2019, The Royal Society of Chemistry.

Qin et al. discovered that the thermoelectric performance of P-type SnSe single crystals can be significantly improved through Te alloying [133]. An exceptionally high *ZT* value of 2.1 at 793 K was reported. Zhang et al. successfully synthesized polycrystalline SnSe_1-*x*_Te*_x_* (*x* = 0.02, 0.03, and 0.05) samples [108]. As shown in Figure 14a–c, the power factor initially increases with pressure, indicating a significant reduction in electrical resistivity. The maximum value of 1 × 10^−4^ Wm^−1^K^−2^ at 3 GPa surpasses other reported values at room temperature [134,135,136]. Su et al. evaluated the influence of pressure on the thermoelectric power factor of Na-doped SnSe crystals up to 2.2 GPa [137]. As shown in Figure 14d–f, the power factor of undoped SnSe and Na-doped SnSe enhancement appears in the low-pressure region. The Na-doped SnSe increases from 33.2 *μ*W cm^−1^K^−2^ at 0.1 GPa to 43.9 *μ*W cm^−1^K^−2^ at 1.925 GPa. The results confirmed that pressure can inherently enhance the thermoelectric properties of SnSe across a broad temperature range within the *Pnma* phase.

**Figure 15:**
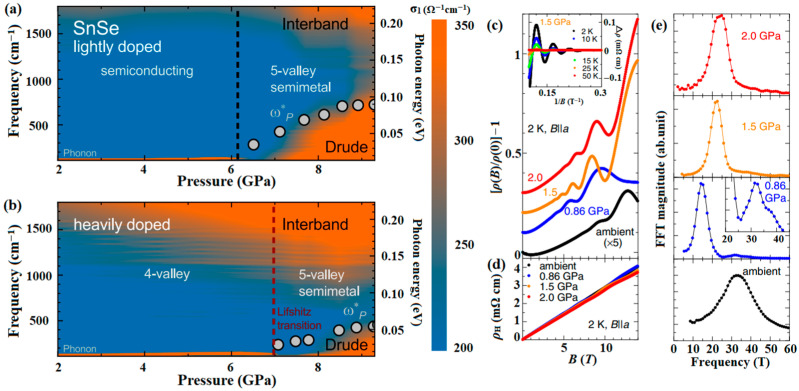
(**a**,**b**) Color maps of the optical conductivity, *σ*_1_(*ω*), as a function of pressure. Reprinted with permission from Ref. [91]. Copyright 2021, Springer Nature. Field (*B*) dependence of (**c**) magneto-resistivity and (**d**) Hall resistivity along the *bc* plane at various pressures at 2 K; (**e**) plots of the fast Fourier transform of the oscillatory part of *ρ* at 2 K at various pressures. Reprinted with permission from Ref. [142]. Copyright 2019, American Physical Society.

As shown in Figure 15a,b, Biesner et al. investigated the semiconductor-semimetal transition in lightly and heavily doped SnSe [91]. It is confirmed that self-doping has a considerable influence on SnSe by shifting the Fermi level, which is more pronounced under applied pressure. Lifshitz transition has been identified as a topological transition in condensed matter physics [138]. Previous investigations of SnSe also detected features at pressures corresponding to the Lifshitz transition [57,62,90,139,140,141]. Nishimura et al. have also reported a marked impact of the pressure-induced Lifshitz transition on thermoelectric performance for moderately hole-doped SnSe [142]. As shown in Figure 15c–e, the field-linear Hall resistivity remains almost unchanged up to 2.0 GPa, implying nearly constant carrier density with pressure. The magneto-resistivity markedly depends on pressure, the observed pressure variation in Shubnikov-de Haas (SdH) oscillations indicates a pressure-induced Lifshitz transition. Furthermore, a new Fermi pocket emerges at around 0.86 GPa, which further evolves in size with increasing pressure. The thermoelectric efficiency enhancement was explained by a multi-valley conductivity resulting from a Lifshitz transition above 1 GPa [91,142].

## 5. Conclusions and Perspectives

Pressure, as a crucial thermodynamic variable, has been employed to alter chemical and crystal structures, induce phase transitions, and thereby impact optical, electronic, and thermoelectric properties. Along with various in situ high-pressure techniques facilitated by DAC, this paper reviewed recent advancements in high-pressure studies of SnSe.

For SnSe bulk materials, two reversible structural transitions have been observed. The first is a second-order transition from *Pnma*-SnSe to *Cmcm*-SnSe, and the second is a first-order transition from *Cmcm*-SnSe to *CsCl*-SnSe. In SnSe nanomaterials, only the first structural transition has been observed, and it takes place at a lower pressure compared to bulk materials. The reduction in transition pressure may be attributed to the volumetric expansion, along with the softening of the Poisson ratio and shear modulus.

High pressure has the fundamental effect of reducing lattice parameters and interatomic distances, which leads to a smaller bandgap. Importantly, after decompression, the bandgap can return to its initial state, signifying that the pressure-induced modulation of the optical bandgap is reversible. Furthermore, pressure also influences the optical in-plane anisotropy of SnSe, which holds promise for the development of new anisotropic optical components, transistors, and photodetectors.

With increasing pressure, the resistivity will decrease significantly, which originates from a metalized phase transition from semiconductor to semi-metal, and the fundamental cause of the change is a structural transition induced by pressure. With further increasing pressure, *CsCl*-SnSe will appear to be a superconductivity phase, but there is disagreement over the pressure range at which superconductivity emerges and the transition temperature.

Pressure can significantly impact the anisotropy of the thermoelectric properties of SnSe. Enhancing the power factor through carrier concentration tuning and reducing thermal conductivity are key strategies to improve the *ZT*. Doping SnSe, due to its low electrical conductivity and the availability of multiple valence band extrema, can result in a higher power factor under high pressure compared to the undoped SnSe.

High pressure opens up avenues for exploring potentially high-performance thermoelectric materials and gaining a comprehensive understanding of the structure and properties of materials under extreme pressure conditions. While significant advancements have been made in this field, using DAC in studying SnSe samples still faces important challenges, and some scientific questions remain unexplored.

First, nanoscience has enabled the preparation of various well-controlled SnSe nanoscale morphologies, such as spherical/faceted nanoparticle, nanosheet, nanoflower, nanotube, and nanowire. Therefore, emerging research lines are expected to explore the influence of key morphological features of SnSe nanostructures, such as the geometry and size of nanocrystals and the facet at the interface between nanomaterials and the surrounding pressure transfer medium. This will involve phase transition behavior and how high pressure behavior affects material properties.

Second, the signal quality in in situ optical measurement using DAC needs to be further improved. Considering the geometry of the DAC and the resulting longer working distance and smaller numerical aperture, optical measurements using the DAC usually have lower signal intensity and signal-to-noise ratio than similar measurements without the DAC. This brings great challenges to some measurements, so the development and refinement of relevant technologies are important for further unleashing the capability of DAC.

Third, the utilization of DAC techniques allows for a more in-depth investigation into the pressure-induced formation of novel low-dimensional structures. Pressure is often used by researchers as a reversible tool to probe material properties; there are instances where the irreversibility of pressure effects can be harnessed to produce innovative structures. The in situ measurement capabilities provided by DAC offer a deeper insight into these processes, which can then be leveraged for designing new materials to create custom structures using pressure as a means to synthesize novel functional materials with unique properties.

Fourth, in addition to the above challenges, there is another problem that needs to be further solved, that is, the theoretical calculation data cannot accurately correspond to the experimental measurement data. This situation is mainly due to the fact that the ideal physical model is embedded in the software program, ignoring some real conditions in the experiments, which makes the high-pressure experiment process difficult to describe.

In summary, high-pressure applications in SnSe face new opportunities and great challenges. In order to clarify these issues, but not be limited to these issues, it would help to understand the physical mechanisms in the modulation of pressure on structure and performance, ultimately improving existing performance or creating higher-performance materials.

## Figures and Tables

**Table 1:** Properties of different pressure-transmitting mediums.

Pressure Transmitting Mediums	Advantages	Limitations	Solidification Pressure (GPa)	Hydrostatic Limit (GPa)	Refs
Methanol/ethanol (4:1) (ME)	Cheap; easy to use	Raman peaks at 432,882 cm^−1^	10.5	20	[47,48]
Methanol/ethanol/water (16:3:1)	Cheap; easy filling	Raman peaks in the range of 0–1000 cm^−1^	14.4	20	[47]
Silicone oil	Cheap; easy to use	Raman peaks at 153,190,490, 687 cm^−1^	0.9	15	[49,50]
Argon	Low Raman background	Difficult to use	1.9	10	[47]
Nitrogen	Low Raman background	Difficult to use	3.0	13	[51]
Helium	Low Raman background; high hydrostatic limit	Difficult to use	12.1	>60	[43]

**Table 2:** Pressure phase transition, volume, bulk modulus, and PTM in SnSe (PTM = Pressure-transmitting media, ME = methanol/ethanol).

Shape	Phase Transition	Pressure (GPa)	Volume (Å^3^)	Bulk Modulus (GPa)	PTM	Ref
bulk	*α*-SnSe to *β*-SnSe	7		*α*-SnSe: 31.48 *β*-SnSe: 40.9		[60]
bulk	*α*-SnSe to *β*-SnSe	10.5	*α*-SnSe: 212.23(5)	*α*-SnSe: 31.1(2)	helium	[61]
nanosheets	*α*-SnSe to *β*-SnSe	6.8	*α*-SnSe: 214.52	*α*-SnSe: 34.4(21) *β*-SnSe: 80.1(11)	4:1 ME	[70]

**Table 3:** Summary of all optical properties of cubic phase π-SnSe at (0, 5, 10, 15, 20, 25, 30, 35 and 40) GPa. Reprinted with permission from Ref. [89]. Copyright 2017 Elsevier.

Property	Pressure Value								
	0 GPa	5 GPa	10 GPa	15 GPa	20 GPa	25 GPa	30 GPa	35 GPa	40 GPa
Static dielectric ε_1_(0)	10.10	17.00	28.20	33.00	42.30	51.10	58.70	67.20	76.20
Screen plasma frequency	8.50 eV	8.84 eV	10.60 eV	10.90 eV	11.40 eV	11.70 eV	12.00 eV	12.30 eV	12.60 eV
Dielectric imaginary part ε_2_(ω) peaks	2.32 eV	1.82 eV	1.78 eV	1.75 eV	1.64 eV	1.54 eV	1.49 eV	1.41 eV	1.35 eV
Refractive index n(0)	3.18	4.12	5.31	5.75	6.51	7.15	7.66	8.20	8.74
Absorption intense peak energy	4.17 eV	4.06 eV	4.35 eV	4.25 eV	4.38 eV	4.45 eV	4.54 eV	4.55 eV	4.58 eV
Absorption peak	1.67 × 10^5^	1.60 × 10^5^	1.97 × 10^5^	2.01 × 10^5^	2.08 × 10^5^	2.13 × 10^5^	2.17 × 10^5^	2.20 × 10^5^	2.24 × 10^5^
Conductivity intense peak energy	2.75 eV	2.54 eV	2.08 eV	2.00 eV	1.97 eV	1.90 eV	1.88 eV	1.78 eV	1.75 eV
Conductivity peak (1/fs)	4.63	4.70	7.68	8.14	8.82	9.34	9.81	10.30	10.80
Reflectivity intense peak energy	6.62 eV	6.97 eV	7.29 eV	7.59 eV	8.03 eV	8.20 eV	8.57 eV	8.95 eV	9.73 eV
Zero frequency coefficient of reflectivity	0.27	0.39	0.46	0.49	0.53	0.57	0.59	0.61	0.63
Loss function	8.49 eV	9.52 eV	10.60 eV	10.90 eV	11.30 eV	11.70 eV	12.00 eV	12.30 eV	12.60 eV

## Data Availability

Not applicable.
